# Fatigue performance in patients with chronic insomnia

**DOI:** 10.3389/fnins.2022.1043262

**Published:** 2022-11-11

**Authors:** Lin Xu, Qianran Zhang, Hongming Dong, Dandan Qiao, Yanyan Liu, Junfang Tian, Rong Xue

**Affiliations:** ^1^Department of Neurology, Tianjin Medical University General Hospital, Tianjin, China; ^2^College of Management and Economics, Tianjin University, Tianjin, China; ^3^Department of Geriatrics, Beijing Luhe Hospital, Capital Medical University, Beijing, China; ^4^Department of Neurology, Liuzhou Workers’ Hospital, Liuzhou, China

**Keywords:** fatigue, task performance and analysis, automobile driving, sleep initiation and maintenance disorders, cognition

## Abstract

Insomnia is associated with fatigue and poor driving performance, thus increasing the risk of traffic accidents. This study aimed to evaluate the effect of fatigue on driving in patients with chronic insomnia in a free-flow traffic scenario and car-following scenario, and to investigate the relationships between driving performance, cognitive function, and insomnia. The Trail Making Test (TMT), Stroop Color and Word Test (SCWT), Symbol Digit Modalities Test (SDMT), and Digit Span Test (DST) of 15 participants with mild-to-moderate chronic insomnia and 16 healthy participants were assessed. During the fatigue driving task, drivers completed simulated driving tasks under free-flow traffic and car-following scenarios. The mean speed (MS), mean acceleration (MA), mean lateral position (MLP), and standard deviation of lateral position (SDLP) were measured to assess driving performance. During fatigued tasks, the MA and MLP in the free-driving scenario were higher than those in the car-following scenario (*P* < 0.01), the SDLP was higher in the insomnia group than in the healthy group (*P* = 0.02), and the interaction effect was significantly different for MLP between the groups (*P* = 0.03). MS was negatively correlated with TMT score, SDMT score, and DST score, and positively correlated with time to complete TMT, errors in SCWT, and time to complete SCWT. SDLP was negatively correlated with DST score and positively correlated with time to complete SCWT. Furthermore, the insomnia group had poorer lateral vehicle control ability than the healthy group. The insomnia group had a more impaired driving performance in the free-driving scenario than in the car-following scenario. Drivers with impaired cognitive function exhibited impaired driving performance.

## Introduction

Adequate sleep is essential for maintaining optimal brain function. People who get insufficient sleep experience a decline in cognitive function, an increase in negative moods, and additional existing burden of diseases (Alhola and Polo-Kantola, 2007; Chuah and Chee, 2008; Chattu et al., 2018). Sleep deficiency includes inadequate sleep duration, irregular sleep timing, poor sleep quality, and sleep disorders, and is extremely common in a modern society (Laposky et al., 2016). As one of the most common sleep disorders, insomnia is characterized by a subjective perception of difficulty in falling asleep and affects approximately 5–30% of the general population (Roth et al., 2011). Insomnia has a negative impact on work performance and productivity (Daley et al., 2009); predisposes patients to a higher risk of medical disorders such as high blood pressure, breathing problems, and chronic pain (Bhaskar et al., 2016): and even impairs quality of life (Mai and Buysse, 2008). Insomnia also causes damage to cognitive function, which is observed as decreased vigilance, memory, attention, and executive functions (Brownlow et al., 2020). Patients with insomnia may develop cognitive impairment that may trigger or worsen insomnia in a cyclic manner, thus aggravating the patient’s condition (Fortier-Brochu and Morin, 2014; Resciniti et al., 2021). Using functional Magnetic Resonance Imaging (fMRI) to analyze brain region activities, studies have revealed that patients with insomnia experience decreased activation of task-appropriate brain regions during N-back tasks, as well as characteristic abnormalities in neural network connectivity related to spatial working memory (Drummond et al., 2013; Li et al., 2016). Furthermore, patients with insomnia are more likely to be involved in traffic accidents. Compared with good sleepers, patients with insomnia face a significantly higher risk of fatigue and car crashes (Leger, 1994; Smolensky et al., 2011) and have three times the risk of having two or three serious accidents compared with normal sleepers (Léger et al., 2006).

Fatigued driving refers to an imbalance in physiological and mental functions during driving, which is objectively manifested as decreased driving functions (Shen et al., 2006). It is a major cause of road traffic accidents. According to the America Insomnia Survey, approximately 15–20% of traffic accidents are reportedly due to fatigue (Roth et al., 2011). Philip et al. (2010) conducted a study of 35,000 drivers and revealed that 31.1% of the drivers had experienced a traffic accident and that 50% of the accidents were related to fatigue. Fatigued driving may be caused by inadequate sleep, a long period of monotonous tasks, health factors like sleep disorders and sleep apnea, etc., and happens when it is hard for a driver to concentrate on the road situation during driving (May and Baldwin, 2009). The neurophysiological signals of sleep-deprived drivers indicate that these drivers experience increased neural activity and cortical activations to overcome fatigue during long-duration driving tasks (Chuang et al., 2018). Driving performance is impaired in fatigued drivers, as demonstrated by longer response times to unexpected changes in the driving environment, a considerable increase in lateral position (Philip et al., 2005a,b; Davenne et al., 2012; Zhao et al., 2012; Zeller et al., 2020), and the ability to make erroneous judgments while driving (Schmidt et al., 2009). Driving performance has been reported to deteriorate further with increased fatigue (Brown, 1997; Leufkens et al., 2009; Herman et al., 2014; Zhang et al., 2016). Insomnia is strongly associated with daytime fatigue and sleepiness, and increases the risk of traffic accidents. Compared with drivers without sleep disorders, drivers with insomnia are more likely to be drowsy, to be involved in serious road crashes, or to fall asleep during driving (Smolensky et al., 2011; Swanson et al., 2012). Results using simulator experiments have proven that after a long-term monotonous driving task, drivers experience impaired driving performance, including increased deviation from speed limits and deviation from absolute speed, reduced ability to maintain constant vehicle velocity, and more inappropriate line crossings (Staner et al., 2005; Perrier et al., 2014; Tsoutsi et al., 2019).

A driving simulator experiment is a frequently used method to evaluate driving performance because data can be easily collected without any risk of real traffic accidents (Sato et al., 2013). Most of the research on driving performance collected data in a free-driving scenario, which means participants were far enough from the leading vehicle in front [e.g., the time headway is more than 2 s (Leufkens et al., 2009)], so that their driving behaviors were not constrained by the leading vehicle in front. Maintaining a steady speed while driving is an example of a situation with free-flowing traffic. However, in real driving scenarios, drivers drive more often with many surrounding cars and have to drive while following a leading vehicle. The following car needs to adjust its speed from time to time to maintain a proper space between it and the leading vehicle. Thus, an individual’s driving behavior in a car-following scenario is different from that in a free-driving scenario (Gipps, 1981; Newell, 2002; Ciuffo et al., 2018). Real-road data have proven that the difference between free-driving and car-following lies in the driving speed and accident rate (Ambros and Kyselý, 2016). No study has investigated the difference in distracted driving performance or effect of insomnia between free-driving and car-following scenarios. This study tried to fill this gap by conducting a simulated driving experiment.

Besides, driving skills and performances are linked to cognitive function. Cognitive functions related to driving process include time perception, attention, visual perception, and reaction time (Sevigny, 2021). Drivers who have deficits in these functions may face driving difficulties while driving. For example, the time to collision in older persons with mild cognitive impairment was considerably shorter than that in healthy controls (Frittelli et al., 2009). Cognitive impairment has a negative impact on driving abilities in old persons or people with multiple sclerosis (Schultheis et al., 2001; Bartolacci et al., 2020). However, few studies have focused on the relationship between cognitive function, driving performance, and insomnia. Therefore, this study tested cognitive performance of insomnia group and healthy group, and investigated the relationship between cognitive function and driving performance.

This study aimed to determine the differences in driving performance between free-driving and car-following driving scenarios, and the effect of insomnia on driving performance. Further, the study aimed to investigate the relationship between driving performance, insomnia, and cognitive function, which includes attention, executive function, and memory. We recruited patients with mild-to-moderate chronic insomnia and healthy control participants to join in this research. They were asked to complete sleep assessments and cognitive function tests. Then, the participants’ performances during fatigued driving scenarios were assessed using a driving simulator. Participants were asked to drive for 20 min in both free-driving and car-following scenarios using the driving simulator. Driving speed, acceleration, and lateral position data during the driving tasks were collected to measure driving performance.

## Materials and methods

### Participants

This study included 15 participants (12 men and 3 women; aged 25–60 years) with mild or moderate chronic insomnia who were treated in the sleep disorder specialist clinic at Tianjin Medical University General Hospital between May 2019 and March 2020. A control group comprising 16 participants (12 men and 4 women), whose age, sex, years of education, and driving experience were matched to the insomnia group participants was also included. Participants in the healthy group were recruited *via* advertisements posted on social media. The participants were asked not to consume alcohol, coffee, strong tea, or other beverages that may affect sleep 1 day before the study. All participants provided informed consent before the study, and it was conducted in accordance with the ethical principles of the Declaration of Helsinki.

Participants with other concomitant sleep disorders, such as sleep apnea syndrome, narcolepsy, restless leg syndrome, or rapid eye movement sleep behavior disorder, were excluded from the study. Participants were also excluded if they had organic heart disease or cardiac arrhythmia, thyroid disease, chronic kidney disease, or a history of mental illnesses (such as severe depression) or neurological disorders (such as stroke, brain tumors, or traumatic brain injury); if they were prone to motion sickness or dizziness; had a history of alcoholism or drug abuse; had less than 3 years of driving experience; or were pregnant or breastfeeding. Participants who experienced shift work or jet lag within a month before participation in the study and those deemed unsuitable for inclusion by the researchers were also excluded. All participants had at least 3 years of driving experience and an average annual mileage of >3,000 km; had at least 9 years of education; did not have reading, writing, or communication disorders; and were right-handed.

Before completing the driving simulator experiments, participants completed sleep assessments including the Pittsburgh Sleep Quality Index (PSQI) (Buysse et al., 1989), Epworth Sleepiness Scale (ESS) (Johns, 1991), and Insomnia Severity Index (ISI) (Bastien et al., 2001). The results given by these questionnaires were assessed by a neurologist to measure participants’ sleep quality. The scales of each questionnaire reflect the participants’ sleep status and can be used as a basis for the diagnosis of insomnia by clinicians. Participants were allocated to the insomnia group (experimental group) or healthy group (control group). Participants in the insomnia group were clinically diagnosed with mild-to-moderate chronic insomnia based on the International Classification of Sleep Disorders–Third Edition (ICSD-3) diagnostic criteria. A PSQI score of ≥7 points and an ISI score of 8–21 points were used to indicate mild-to-moderate chronic insomnia. All patients with insomnia had a history of using non-benzodiazepines, and they were untreated or had a treatment-free withdrawal period for at least 2 weeks. The healthy group comprised participants who had a normal sleep status, did not meet the ICSD-3 diagnostic criteria for chronic insomnia, had a PSQI score of <7 points, and had an ISI score of ≤7 points.

### Assessment of cognitive function test

A series of cognitive function tests were conducted to measure the abilities that may affect driving performance, including attention, processing speed, general cognitive function, executive function, and memory.

#### Trail Making Test

The Trail Making Tests (TMTs) are popular neuropsychological instruments to measure attention and executive ability and includes the TMT-A and TMT-B (Bowie and Harvey, 2006). The TMT-A requires participants to connect a set of numbers in sequential order. In the TMT-B, the numbers are enclosed in circles and squares, and the participants are required to connect the numbers in sequential order while alternating between a circle and a square without lifting the pen from the paper. The time to complete the entire test and number of correctly connected numbers were recorded in this study.

#### Stroop Color and Word Test

The Stroop Color and Word Exam (SCWT) is a neuropsychological test that is often used to measure cognitive interference inhibition, and it evaluates selective attention and executive function (Law et al., 2014). In this study, the SCWT was performed using three cards. The participants were asked to name the colors of circles on card A and colors of Chinese characters on cards B and C (each character represented a different color). The time taken to complete the test and number of errors made were recorded.

#### Symbol Digit Modalities Test

The Symbol Digit Modalities Test (SDMT) is a screening test used to identify neurological impairment in clinical and research settings and measures speed of processing and executive ability (Smith, 1973). Participants were allotted 90 s to pair numbers with geometric figures using a reference key. Lower scores indicate poorer attention and slower visual scan speeds. The score of SDMT was represented by the number of correctly paired figures.

#### Digit Span Test

The Digit Span test (DST) is one of the most regularly used tests of immediate language recall, attentional ability, and working memory in neuropsychological research and clinical examinations (Blackburn and Benton, 1957). A sequence of numbers was read to each participant in this study. The participant then repeated the numbers in the order that they were read and in reverse order. The DST score was represented by the length of the longest correctly repeated sequence.

### Driving simulator experiments

**FIGURE 1:**
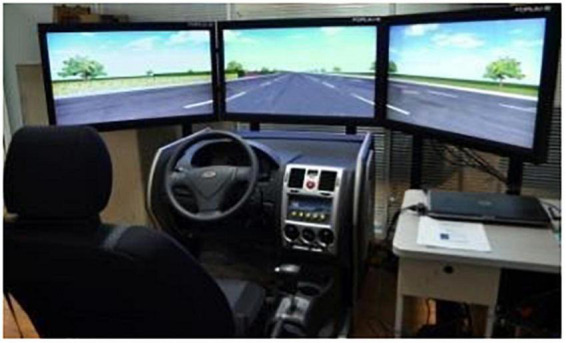
The FORUM8 driving simulator used in this experiment.

The FORUM8 driving simulator (FORUM8 Co., Ltd., Japan) used in this experiment is shown in Figure 1. The simulator is equipped with three 42-inch display monitors, which displayed simulated driving scenarios from the left front view, middle front view, and right front view. UCwin/Road software was used to construct the simulation scenario and to log driving data of the simulated driving vehicles throughout the experiment. Participants underwent a brief training session of approximately 10 min to familiarize themselves with the simulator controls and driving environment.

#### Fatigued driving task

The fatigued driving task comprised two scenarios: one without a leading vehicle (free-driving scenario) and one with a leading vehicle (car-following scenario). The participants completed the driving tasks in the two scenarios in a random order.

**FIGURE 2:**
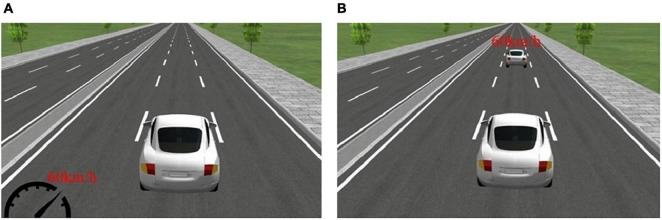
Scenarios for the fatigued driving experiment. **(A)** The free-driving fatigued driving scenario and **(B)** car-following fatigued driving scenario are shown.

The free-driving scenario included a straight, three-lane road with the vehicle traveling in the middle lane throughout the entire scenario. The participants were required to keep driving without virtual surrounding vehicles and while maintaining a driving speed of 60 km/h for 20 min to simulate fatigued driving under monotonous driving conditions (Figure 2A). The car-following scenario was identical to the free-driving scenario, except for the presence of a leading vehicle traveling at 60 km/h in front. Participants were asked to follow the leading vehicle at a certain following distance without colliding with or losing sight of the leading vehicle (Figure 2B). The 20-min scenario simulated fatigued driving under car-following conditions. During both fatigued driving scenarios, the mean speed (MS), mean acceleration (MA), mean lateral position (MLP), and standard deviation of lateral position (SDLP) were used to evaluate the driving performance during simulated driving. Driving performance data was collected automatically by UCwin/Road software.

The entire driving simulator experiment was completed in approximately 100 min. Participants first completed a 10-min training session to learn the basic operation of the driving simulator. After the training session, the participants completed a 70-min distracted driving task (unrelated with this study) and the 20-min fatigued driving task. Studies have confirmed that 40–60 min of simulated driving is sufficient to induce driving fatigue (Zeller et al., 2020). In this study, participants completed approximately 70 min of driving simulation testing before the start of the fatigued driving task, and were therefore in a state of fatigue. To ensure that the participants were fully acclimated to the simulated driving scenarios, data from the second half of the fatigued driving task (from 10 min after the start of the experiment to the end of the experiment) were used in the analyses.

### Statistical analyses

The correlations of sleep quality with cognitive function and driving performance parameters were assessed using the pooled data of all participants. Statistical analyses were performed using SPSS 23.0 software.

Results of all data were expressed as means ± standard deviations. Chi-square (χ^2^) statistics were used to measure the difference in sex, age, and years of education between the insomnia and healthy groups. The Mann–Whitney *U* test was used to compare sleep quality and cognitive function. To analyze velocity, acceleration, and lateral position data during the fatigued driving task, we used two-way analysis of variance (ANOVA) where group was regarded as the between-subjects factor and leading-vehicle (free-driving or car-following) was regarded as the within-subjects factor. Spearman’s analysis was used to assess correlations. A *P*-value ≤0.05 was considered statistically significant.

## Results

### Sleep quality and cognitive function test

The participants’ socio-demographic characteristics (age, sex, years of education, and driving experience) are described in Table 1. Most of the participants were aged between 35 and 45 years, with more than 10 years of education and driving licenses for more than 5 years. Most of the participants were male. There were no significant differences in socio-demographic characteristics between the groups. Regarding sleep quality, patients with insomnia were more likely to have sleep disorders (PSQI > 7), sleepiness (ESS > 6), or insomnia (ISI > 7). Normal sleepers did not show signs or symptoms of the disorders. The PSQI, ESS, and ISI scores were significantly lower in the healthy group than in the insomnia group (Table 1).

**TABLE 1:** Means (±SD) of participant characteristics.

Characteristic	Insomnia group Mean ± SD	Healthy group Mean ± SD	χ^2^/*Z*	*P*
Age (years)	41.20 ± 11.66	40.31 ± 12.77	126.5	0.80
Sex (male/female)	12/3	12/4	0.11	0.74
Years of education (years)	15.47 ± 2.75	15.81 ± 3.10	1.21	0.30
Driving experience (years)	11.20 ± 8.29	12.38 ± 10.05	114.5	0.83
PSQI	10.20 ± 2.91	1.69 ± 1.35	240	0.01**
ESS	8.20 ± 5.32	3.00 ± 3.20	187	0.01**
ISI	11.07 ± 3.90	1.13 ± 1.45	240	<0.01**

**p < 0.01. PSQI, Pittsburgh Sleep Quality Index; ESS, Epworth Sleepiness Scale; ISI, Insomnia Severity Index.

The results of cognitive function testing are shown in Table 2. There was no significant difference in TMT and SDMT scores between the two groups. The mean and standard deviation (SD) of time to complete TMT and SCWT were higher in the insomnia group than in the healthy group, though not significantly (Mann–Whitney *U* test, *P* > 0.05). The mean and SD of the number of errors in SCWT were also higher in the insomnia group than in the healthy group, though not significantly (Mann–Whitney *U* test, *P* > 0.05). Finally, the insomnia group had a significantly lower DST score than the control group (Mann–Whitney *U* test, *P* = 0.02).

**TABLE 2:** Cognitive function test measures for groups.

	Insomnia group Mean ± SD	Healthy group Mean ± SD	*Z*	*P*
TMT				
Score (points)	42.67 ± 4.47	41.56 ± 4.34	135.5	0.545
Time (s)	130.24 ± 36.73	119.43 ± 22.51	130	0.711
SCWT				
Errors	0.87 ± 2.03	0.13 ± 0.34	132	0.654
Time (s)	62.55 ± 24.57	51.02 ± 7.71	148	0.281
SDMT score (points)	56.00 ± 13.43	58.38 ± 9.79	113.5	0.800
DST score (points)	15.00 ± 0.83	17.06 ± 2.11	62.5	0.021*

*p < 0.05. TMT, Trail Making Test; SCWT, Stroop Color and Word Test; SDMT, Symbol Digit Modalities Test; DST, Digit Span Test.

### Driving experiments

**FIGURE 3:**
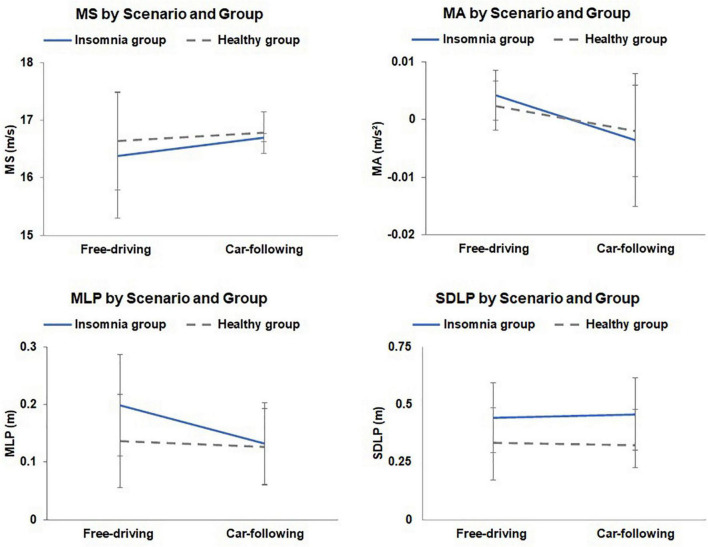
The mean speed (MS), mean acceleration (MA), mean lateral position (MLP), and standard deviation of lateral position (SDLP) throughout the fatigued driving task for each group. Error bars represent the standard errors of the means.

A two-way ANOVA was conducted to assess the effect of driving performance on each group (insomnia group and control group) and the leading vehicle (free-driving scenario and car-following scenario). Figure 3 displays the means and standard deviations of the fatigued driving task, and Table 3 demonstrates the ANOVA results. The MA and MLP were significantly higher in the free-driving scenario than in the car-following scenario (*P* < 0.01). The SDLP was significantly lower in the healthy group than in the insomnia group (*P* = 0.02). No significant group effect was found in other parameters. The interaction effects of the leading-vehicle design and grouping was significantly different for MLP (*P* = 0.03).

**TABLE 3:** Fatigued driving task results.

	Group effect		Leading vehicle effect		Interactive effect	
			
	*F*	*P*	*F*	*P*	*F*	*P*
MS (m/s)	0.86	0.36	1.56	0.22	0.23	0.64
MA (m/s^2^)	0.01	0.94	10.22	<0.01**	0.80	0.38
MLP (m)	1.53	0.23	10.62	<0.01**	5.65	0.03*
SDLP (m)	6.30	0.02*	0.01	0.91	0.35	0.56

*p < 0.05; **p < 0.01. MS, mean speed; MA, mean acceleration; MLP, mean lateral position; SDLP, standard deviation of lateral position.

### Correlation between driving performance and cognitive function

Table 4 displays the correlations between cognitive test measurements and driving performances. The TMT score was negatively correlated with the MS in the free-driving scenario (ρ = −0.42, *P* = 0.02), and the time taken to complete the TMT was positively correlated with the MS in the free-driving scenario (ρ = 0.41, *P* = 0.02). The number of errors in the SCWT was positively correlated with the MS in the car-following scenario (ρ = 0.39, *P* = 0.03). The time taken to complete the SCWT was positively correlated with the MS (ρ = 0.48, *P* = 0.01) and SDLP (ρ = 0.47, *P* < 0.01) in the free-driving scenario, and was also positively correlated with the MS (ρ = 0.53, *P* < 0.01) and SDLP (ρ = 0.47, *P* = 0.01) in the car-following scenario. The SDMT score was negatively correlated with the MS (ρ = −0.50, *P* < 0.01) and in the free-driving scenario. The DST score was negatively correlated with the MS (ρ = −0.37, *P* = 0.04) and SDLP (ρ = −0.39, *P* = 0.03) in the car-following scenario.

**TABLE 4:** Correlations between cognitive function and fatigued driving performance.

		Free-driving scenario		Car-following scenario	
			
		ρ	*P*	ρ	*P*
TMT score	MS	−0.42	0.02*	−0.14	0.45
	MA	−0.05	0.79	−0.13	0.49
	MLP	0.09	0.64	0.03	0.86
	SDLP	0.02	0.91	−0.03	0.87
Time to complete TMT	MS	0.41	0.02*	0.10	0.58
	MA	−0.11	0.55	0.28	0.13
	MLP	0.07	0.72	0.05	0.79
	SDLP	0.02	0.94	0.05	0.80
Errors in SCWT	MS	0.18	0.34	0.39	0.03*
	MA	−0.01	0.95	0.24	0.20
	MLP	0.30	0.10	0.20	0.30
	SDLP	0.19	0.31	0.13	0.49
Time to complete SCWT	MS	0.48	0.01**	0.53	<0.01**
	MA	−0.09	0.64	0.15	0.41
	MLP	0.32	0.08	0.23	0.22
	SDLP	0.47	0.01**	0.47	0.01**
SDMT score	MS	−0.50	<0.01**	−0.18	0.33
	MA	0.07	0.70	−0.01	0.98
	MLP	−0.26	0.50	−0.21	0.50
	SDLP	−0.13	0.49	−0.22	0.25
DST score	MS	−0.16	0.40	−0.37	0.04*
	MA	−0.26	0.16	−0.11	0.54
	MLP	−0.15	0.43	−0.13	0.50
	SDLP	−0.28	0.12	−0.39	0.03*

*p < 0.05; **p < 0.01. TMT, Trail Making Test; SCWT, Stroop Color and Word Test; SDMT, Symbol Digit Modalities Test; DST, Digit Span Test; MS, mean speed; MA, mean acceleration; MLP, mean lateral position; SDLP, standard deviation of lateral position.

## Discussion

This study aimed to investigate the fatigued driving performance of patients with mild-to-moderate insomnia in comparison with normal sleepers in free-driving and car-following scenarios. Our results revealed that drivers with insomnia had poorer lateral vehicle control ability than healthy drivers under fatigue. The driving behavior of both groups of drivers was different between traffic states, and the ability of longitudinal and lateral vehicle control was more impaired in the free-driving scenario than in the car-following scenario. Drivers with impaired cognitive function had an impaired driving performance.

Except for DST (Mann–Whitney *U* test, *P* = 0.02), most of the cognitive function tests were not significantly different between the insomnia group and healthy group (Table 2). The length of numbers that the insomnia drivers were able to remember was shorter than that in the healthy group, indicating that the insomnia participants had impairments in working memory. The differences in other cognitive function tests were not significant, probably because the participants we recruited were patients with mild-to-moderate chronic insomnia. These patients had milder symptoms and less pronounced cognitive impairment compared to those with severe chronic insomnia. In a study by Fortier-Brochu et al. (2012), they obtained similar conclusions in a larger sample: they observed impairments in working memory in patients with mild-to-moderate insomnia, while general cognitive functions, language functions, and attention were not significantly different. Therefore, the observation of a slight cognitive impairment in the insomnia group compared to the healthy group is reasonable and consistent with previous research.

Next, we focused on the driving performance of the simulated driving task. MS and MA describe the driver’s ability in maintaining stable longitudinal movement. The MS of drivers in both groups was maintained between 16 and 17 m/s under both free-driving and car-following scenarios, which is consistent with the driving task requiring drivers to drive at 60 km/h (16.7 m/s). This indicates that all participants followed the experimental instructions carefully and completed the driving task. The MA did not vary significantly between drivers, which reflects that the driving ability in longitudinal vehicle control was not influenced by groups. Insomnia did not affect the driver’s speed control ability. However, the MA was lower in the car-following scenario than in the free-driving scenario. We speculate that this may be due to the fact that when a leading vehicle is present, drivers might have to maintain a more constant speed by reducing acceleration to maintain relatively safe time headway. The results of MS and MA revealed that insomnia did not affect the driver’s longitudinal vehicle control. Their driving performance differed between the free-driving and car-following states, regardless of whether the driver had insomnia or not. Furthermore, the drivers had a more stable driving ability during acceleration when following a leading vehicle.

Mean lateral position and SDLP are used to describe the drivers’ ability in maintaining stable lateral movement. MLP measures the distance of deviation from the middle lane by the vehicle. The leading vehicle effect and interaction effect were significantly different. Figure 3 showed that the MLP in the free-driving scenario was higher than that in the car-following scenario for both the insomnia and healthy groups, implying that drivers were less likely to deviate from the middle of the lane when driving after a leading vehicle. Similar to the MA analysis, we speculate that this is because drivers might take the leading car as a reference, and are therefore able to maintain more stable lateral vehicle control. Additionally, the MLP of the insomnia group in the free-driving scenario was significantly higher than that in the car-following scenario, and was also higher than the MLP of healthy drivers in both scenarios, suggesting that insomnia impaired driving ability in lateral vehicle control. Therefore, drivers with insomnia had a difficulty staying in the middle of the driving lane. If a leading vehicle exists, drivers with insomnia are able to take the leading vehicle as a reference for following, which explains the significantly lower MLP in the car-following scenario. Moreover, the SDLP results suggest that there was a significant group effect, with drivers with insomnia having higher values in the presence or absence of the leading car. This indicates that drivers with insomnia cannot maintain steady control of lateral movement during fatigued driving, resulting in frequent swaying of the vehicle from side to side. The results of MLP and SDLP indicate that drivers with insomnia have a certain degree of safety risk while driving. If drivers with insomnia take a drive, they are better off driving their vehicles in a car-following state, to reduce the risk of driving out-of-lane.

Finally, this study compared the relationships between cognitive function and fatigued driving performance. According to the results of the correlation analysis (Table 4), drivers with impaired cognition (low test scores, long time to complete tests, and more errors in test) drove with a higher MS in the task; faster speeds may indicate higher safety risks. Moreover, drivers with a long time to complete SCWT and low DST scores had a higher SDLP in driving, indicating that drivers with impairments in executive function and working memory may face more difficulties in maintaining the vehicle in a lateral position. Combined with the cognitive function and driving performance differences between the groups, we hypothesized that the deficits in driving performance of drivers suffering from insomnia may be related to their impaired cognitive function.

This study had some limitations. We included a small sample size, which may limit our findings’ generalizability to large samples. Additional studies with larger sample sizes are needed to support this hypothesis. Moreover, the differences in driving performance between patients with insomnia and normal sleepers when following a leading vehicle need to be explored in future studies. The results of this study are consistent with those of existing literature in suggesting that patients with insomnia may experience impairment of sustained attention and reaction ability; however, the study’s sample size was again too small to produce statistically significant data. To obtain significant statistical results, the experimental sample size of future studies should be at least 90 participants, and a more in-depth analysis of driving performance in patients with mild-to-moderate chronic insomnia compared with that of normal sleepers should be performed (Perrier et al., 2014). Secondly, the two participant groups were highly related to each other, but multiplicity adjustments were not taken into account in the analyzing trials, which refers to a potential increase in Type I error. Moreover, experimental data of patients with severe chronic insomnia was not collected because we could not recruit enough volunteers. Further research should focus on the driving performance and neuropsychological functions of these patients.

To conclude, drivers with mild-to-moderate chronic insomnia had difficulty maintaining their driving performance when fatigued, and that these drivers may experience increased safety risks. In a free-driving fatigued task, patients with insomnia have impaired abilities in lateral position vehicle control than healthy drivers. For car-following driving tasks, the leading vehicle can be regarded as a reference to help drivers reduce the number of lateral positions caused by fatigue, especially for drivers with insomnia. Cognitive abilities also have an impact on driving performance. Drivers with a lower cognitive function may exhibit a higher driving speed and higher SDLP. The results of this study will help doctors to guide the driving behaviors of patients with insomnia and serve as a reference for future investigations on cognitive decline and driving performances in patients with insomnia.

## Data availability statement

The raw data supporting the conclusions of this article will be made available by the authors, without undue reservation.

## Ethics statement

The studies involving human participants were reviewed and approved by the Medical Ethics Committee of Tianjin Medical University General Hospital. The patients/participants provided their written informed consent to participate in this study.

## Author contributions

JT and HD conceived of the presented idea. DQ and YL carried out the experiment. LX and QZ performed the analysis, drafted the manuscript, and designed the figures. RX supervised the findings of this work. All authors discussed the results and contributed to the final manuscript.
